# A Diagnostic Pitfall: Carbamazepine-Induced Drug Reaction With Eosinophilia and Systemic Symptoms (DRESS) Syndrome Masquerading As Angiotensin-Converting Enzyme Inhibitor Angioedema

**DOI:** 10.7759/cureus.105009

**Published:** 2026-03-10

**Authors:** Roberto R Gonzalez Alvarez

**Affiliations:** 1 Internal Medicine, Cape Coral Hospital, Cape Coral, USA

**Keywords:** carbamazepine, corticosteroids, dress syndrome, drug allergy, drug reaction with eosinophilia and systemic symptoms, eosinophilia, hypersensitivity

## Abstract

Drug reaction with eosinophilia and systemic symptoms (DRESS), also known as drug-induced hypersensitivity syndrome (DIHS), is a severe, multiorgan, delayed hypersensitivity reaction with significant morbidity. Diagnosis is often delayed due to its mimicry of other conditions. We present the case of a 69-year-old Hispanic male with hypertension and stage two chronic kidney disease who developed trigeminal neuralgia and was started on carbamazepine. Approximately seven weeks later, he presented with a generalized rash, angioedema, and mucosal swelling, initially attributed to his concurrent lisinopril therapy. Despite discontinuation of lisinopril and treatment with corticosteroids and antihistamines, his condition progressed with new systemic symptoms, including paresthesia, dysgeusia, polydipsia, polyuria, and weight loss. The progression of symptoms despite this intervention was the pivotal diagnostic clue. Hospital admission revealed marked eosinophilia (8.9 percent), mild transaminitis, and a diffuse erythrodermic rash involving more than 90 percent of his body surface area. A diagnosis of probable DRESS syndrome (RegiSCAR score six) secondary to carbamazepine was made. Immediate discontinuation of carbamazepine and initiation of intravenous corticosteroids led to rapid clinical and laboratory improvement. He was discharged on a prolonged oral steroid taper with complete resolution at follow-up. This case underscores the critical importance of considering DRESS in any patient with a rash and systemic symptoms occurring two to eight weeks after initiation of a high-risk drug, even in the presence of other potential culprits. It highlights that clinical progression despite withdrawal of one suspected agent should prompt urgent re-evaluation for an alternative etiology, such as DRESS.

## Introduction

Drug reaction with eosinophilia and systemic symptoms (DRESS), or drug-induced hypersensitivity syndrome (DIHS), represents a severe, idiosyncratic, T-cell-mediated drug reaction. It is characterized by a constellation of clinical findings, including cutaneous eruption, hematologic abnormalities (eosinophilia and/or atypical lymphocytes), fever, lymphadenopathy, and internal organ involvement (most commonly liver, kidney, and lungs) [1,2]. With an estimated mortality rate of up to 10-20%, early recognition and prompt withdrawal of the offending agent are paramount [3, 4].

The pathogenesis is complex, involving genetic predisposition (e.g., specific HLA alleles), altered drug metabolism, and viral reactivation (particularly human herpesvirus-6, HHV-6) [5]. A hallmark of DRESS is its delayed onset, typically emerging two to eight weeks after initiation of the culprit medication [6]. Common inciting drugs include aromatic antiepileptics (e.g., carbamazepine, phenytoin), allopurinol, sulfonamides, and minocycline [7].

Diagnosis relies on clinical criteria, with the RegiSCAR scoring system being the most widely used validation tool [8]. We present a classic yet diagnostically challenging case of carbamazepine-induced DRESS that was initially mistaken for angiotensin-converting enzyme inhibitor (ACEi)-induced angioedema, emphasizing a critical pitfall in clinical management.

DRESS must be distinguished from other conditions, such as viral exanthems, sepsis, Stevens-Johnson syndrome, and other drug reactions, including ACE-inhibitor-induced angioedema, which was the initial diagnostic consideration in this case.

## Case presentation

A 69-year-old Hispanic male with a past medical history of essential hypertension, dyslipidemia, and stage two chronic kidney disease (baseline eGFR 65 mL/min/1.73m²) presented to the emergency department on January 3, 2026, with a progressive generalized rash and systemic symptoms. His home medications had included lisinopril, hydralazine, and atorvastatin for several years. For the new onset of idiopathic trigeminal neuralgia diagnosed by his primary care physician, he was initiated on carbamazepine extended-release (100 mg orally twice daily) on November 10, 2025.

Approximately seven weeks later, on December 29, 2025, he developed an acute generalized rash, facial angioedema, and mucosal swelling. At an outpatient visit, this reaction was attributed to lisinopril. Consequently, lisinopril was discontinued and replaced with losartan. He was treated with intramuscular triamcinolone acetonide (80 mg), a short course of oral prednisone (10 mg daily for eight days on a taper), and fexofenadine. Notably, carbamazepine was continued.

His condition worsened over the subsequent five days. Upon presentation to our facility, he reported progression of the rash along with new symptoms: numbness of the tongue with loss of taste (dysgeusia), "pins and needles" sensation in his feet, subjective hand swelling, profound thirst with polyuria (necessitating waking three to four times nightly), decreased appetite, and an unintentional weight loss of four pounds. He denied fever, chills, or respiratory symptoms.

**Figure 1:**
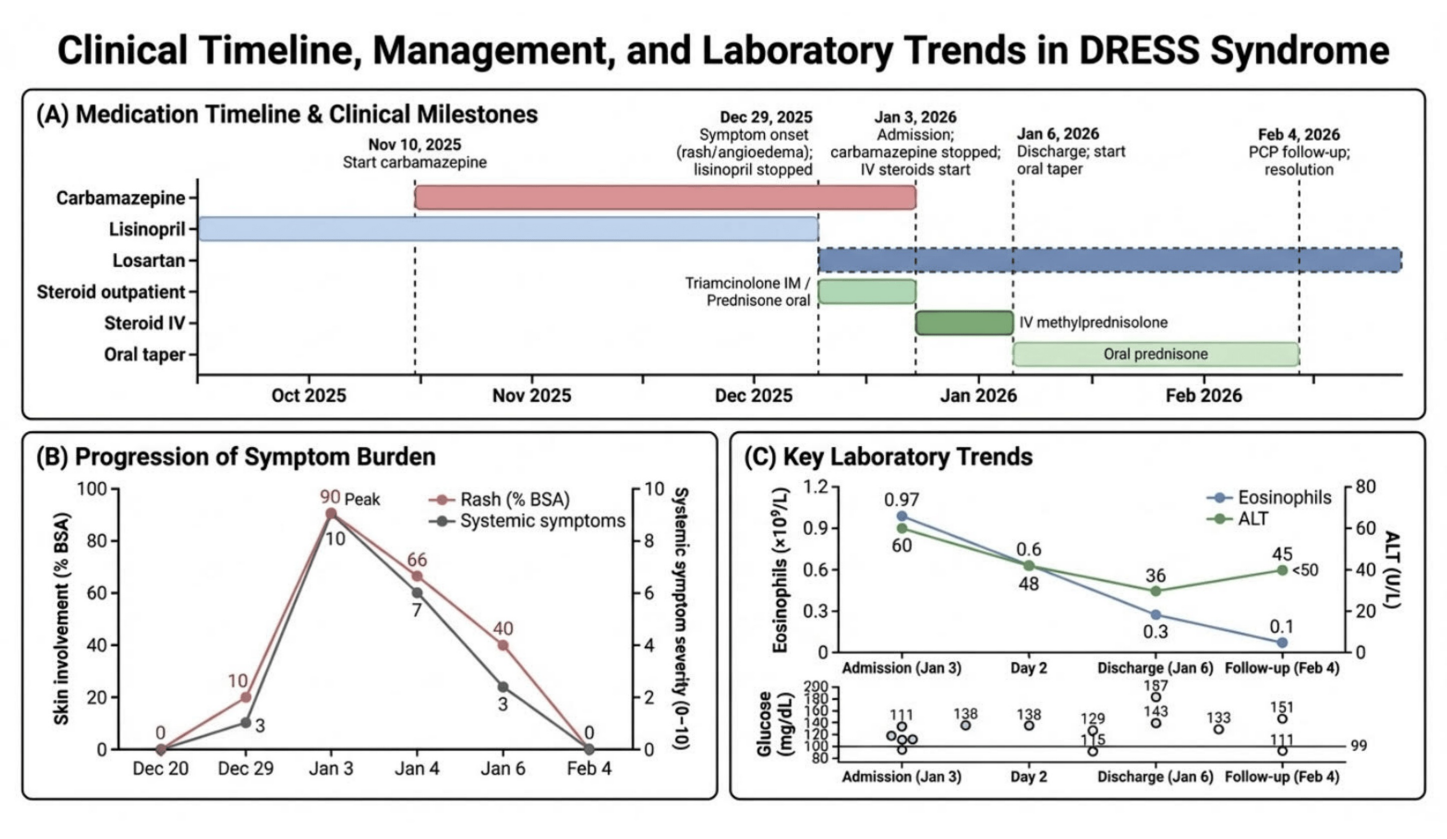
Clinical course, management, and laboratory trends in drug reaction with eosinophilia and systemic symptoms (DRESS) syndrome (A) Medication timeline and critical clinical events. The horizontal bars illustrate the administration periods of key medications: the culprit drug (carbamazepine), the initially suspected drug (lisinopril), its replacement (losartan), and systemic corticosteroid courses. Vertical markers indicate major clinical decision points. (B) Progression of symptom burden. The line graph tracks the estimated extent of skin involvement (percentage of body surface area, % BSA) and the severity of systemic symptoms (e.g., mucosal involvement, paresthesia, constitutional symptoms) over time, demonstrating peak severity at hospital admission and rapid improvement following intervention. (C) Key laboratory trends. This panel shows the trajectory of primary laboratory abnormalities: absolute eosinophil count (hematologic hallmark), alanine aminotransferase (ALT, indicating hepatic involvement), and point-of-care blood glucose levels (reflecting endocrine/metabolic disturbance) throughout the hospitalization.

The clinical chronology, highlighting the critical delay in identifying the correct culprit drug, is summarized in Figure 1.

**Figure 2:**
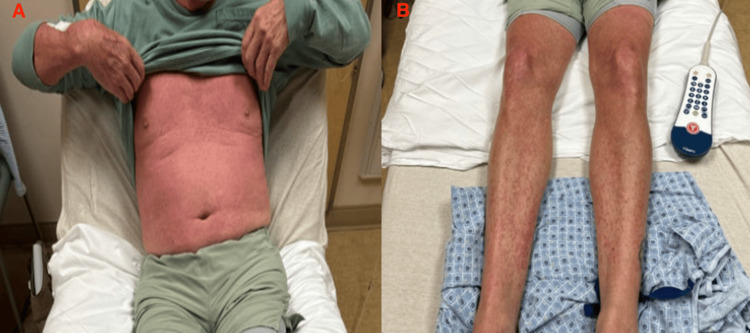
Diffuse erythrodermic rash on admission (A) Clinical photograph of the patient's upper extremities, chest, and abdomen. (B) Clinical photograph of the patient's bilateral lower extremities. Both images demonstrate the confluent, erythematous rash involving >90% of the body surface area, consistent with the cutaneous presentation of drug reaction with eosinophilia and systemic symptoms (DRESS) syndrome.

On examination, he was afebrile, alert, and oriented. Vital signs were stable, with asymptomatic bradycardia noted. He had a history of asymptomatic bradycardia (heart rate 50-55 bpm) documented on prior outpatient ECGs, with no acute ischemic changes or conduction abnormalities noted during admission. Dermatological examination revealed a diffuse, intensely erythematous (erythrodermic) rash. The rash was intensely pruritic, morbilliform, and confluent, beginning on the trunk and spreading centrifugally, involving over 90 percent of his body surface area (Figure 2), with notable involvement of the oral mucosa (buccal and lingual erythema) and genital mucosa (scrotal erythema and edema). Mild facial edema was present. Cardiopulmonary auscultation revealed bibasilar fine crackles. A chest radiograph performed on admission revealed no cardiomegaly, pulmonary edema, or infiltrates, making heart failure an unlikely cause of the auscultated crackles.

Initial diagnostic laboratory results are summarized in Table 1. Urinalysis was unremarkable.

**Table 1:** Key laboratory findings on hospital admission The table summarizes the initial diagnostic laboratory results supporting the diagnosis of drug reaction with eosinophilia and systemic symptoms (DRESS) syndrome. Hyperglycemia was noted across multiple point-of-care checks during the initial admission period. For virologic studies, results below the listed quantitative thresholds are reported as "Not detected" or "Negative", indicating no evidence of active viral reactivation or prior infection relevant to DRESS pathogenesis. PCR: Polymerase Chain Reaction; VCA IgG: Viral Capsid Antigen Immunoglobulin G; IgG: Immunoglobulin G.

Parameter (unit)	Patient value	Reference range / negative threshold	Interpretation
Complete blood count			
White blood cell count (x 10⁹/L)	10.9	3.8 - 10.6	Leukocytosis
Absolute eosinophil count (x 10⁹/L)	0.97	0.0 - 0.5	Marked eosinophilia
Eosinophils (%)	8.9	0.0 - 6.0	Marked eosinophilia
Hepatic function panel			
Alanine aminotransferase - ALT (U/L)	60	< 50	Mild transaminitis
Renal function panel			
Serum creatinine (mg/dL)	0.83	0.7 - 1.3	No acute kidney injury
Point-of-care glucose			
Random glucose (mg/dL)	111 - 187	65 - 99	Hyperglycemic range
Therapeutic drug monitoring			
Carbamazepine level (µg/mL)	5.3	4.0 - 12	Therapeutic range
Virologic studies			
Human herpesvirus-6 (HHV-6) quantitative PCR (copies/mL)	Not Detected	< 500	No active viremia
Human herpesvirus-6 (HHV-6) IgG serology	Not Detected	< 1:10 Titer	Antibody not detected
Epstein-Barr virus (EBV) quantitative PCR (IU/mL)	Not Detected	< 35.0	No active viremia
Epstein-Barr virus (EBV) VCA IgG serology (U/mL)	Negative	< 18.0	Negative past exposure
Cytomegalovirus (CMV) quantitative PCR (IU/mL)	Not Detected	< 34.5	No active viremia
Cytomegalovirus (CMV) IgG serology (U/mL)	Negative	< 0.60	Negative past exposure

Application of the RegiSCAR scoring system yielded a score of five, indicating "probable DRESS" [8]. Points were assigned for: rash more than 50 percent body surface area (two points), eosinophilia more than 0.7 x 10⁹/L (one point), hepatic involvement (alanine aminotransferase more than two times the upper limit of normal) (one point), and pulmonary involvement (bibasilar crackles) (one point). The RegiSCAR scores can be seen in Table 2.

**Table 2:** RegiSCAR scoring system applied to this case Scoring system adapted from Kardaun et al., 2013 [8]. Interpretation: Score 2-3 = Possible; 4-5 = Probable; ≥6 = Definite DRESS. This case represents Probable DRESS. DRESS - drug reaction with eosinophilia and systemic symptoms; ALT - alanine aminotransferase

RegiSCAR criterion	Patient finding	Points
Fever ≥38.5°C	Absent (possibly masked by prior steroids)	0
Lymphadenopathy	Not detected	0
Eosinophilia	≥0.7 x10⁹/L and <1.5 x10⁹/L (0.97 x10⁹/L)	1
Atypical lymphocytes	Not detected	0
Skin rash extent	Rash >50% body surface area	1
Skin rash suggestive of DRESS	Yes (erythroderma, facial edema, purpura)	1
Skin biopsy suggestive of DRESS	Not performed	0
Internal organ involvement	Liver (ALT 60 U/L), Lung (bibasilar crackles)	1
Resolution ≥15 days	Yes	0
Evaluation for other causes	Negative viral studies, no alternative diagnosis	0
Total score		5

The diagnosis of carbamazepine-induced DRESS syndrome was established. Carbamazepine was permanently discontinued on admission. Management consisted of intravenous methylprednisolone (125 mg initial dose, followed by 40 mg every eight hours), topical clobetasol ointment, and supportive care with intravenous hydration. Diphenhydramine was administered as needed for pruritus.

The patient demonstrated a rapid and favorable response. By hospital day two, the rash involved approximately 66 percent of his body surface area. By day three, it had receded to 40 percent, with complete resolution of mucosal involvement. His eosinophil counts normalized, and alanine aminotransferase (ALT) decreased to 36 U/L. He was discharged on hospital day three (January 6, 2026) on a prolonged, 42-day oral prednisone taper to prevent relapse.

At a follow-up visit on February 4, 2026, the patient reported complete resolution of all symptoms. Physical examination was normal, and repeat laboratory studies showed normalization of the complete blood count, liver function tests, and glucose levels.

## Discussion

This case provides several crucial learning points regarding the diagnosis and management of DRESS syndrome. First, it illustrates a common diagnostic pitfall: the premature attribution of a drug reaction to the most common or obvious culprit, in this instance, ACEi-induced angioedema [9]. While this initial diagnosis was plausible, the subsequent clinical course was the key diagnostic clue. The progression of systemic symptoms despite the withdrawal of lisinopril and administration of corticosteroids mandated a search for an alternative, ongoing process. This pitfall and the classic latency period are visually emphasized in the clinical timeline (Figure 1).

Second, the timeline was classic for DRESS, with symptom onset approximately seven weeks after initiating carbamazepine, aligning perfectly with the known two-to-eight-week latency period [6]. This temporal relationship is a cornerstone of diagnosis, but is often overlooked if the medication history is not meticulously reviewed with this specific window in mind.

Third, the presentation highlighted the multisystem nature of DRESS. Beyond the cutaneous and hematologic manifestations, the patient exhibited potential neurological (paresthesia, dysgeusia), endocrine (polydipsia/polyuria, hyperglycemia), and pulmonary (bibasilar crackles) involvement. Hyperglycemia is a recognized complication that may arise from the systemic inflammatory state of DRESS itself, but in this patient, it was likely multifactorial, attributable to both the systemic inflammatory state of DRESS and the recent high-dose corticosteroid therapy [10]. The absence of fever and negative viral serologies, while not typical, does not exclude the diagnosis, as not all cases demonstrate these features [8]. The absence of fever may be attributable to the prior corticosteroid administration during the outpatient evaluation.

Fourth, management principles were effectively demonstrated. The cornerstone of therapy is immediate and permanent discontinuation of the culprit drug [11]. The use of systemic corticosteroids is standard for moderate-to-severe cases to suppress the immune-mediated organ injury [2,4,12]. The successful response to intravenous steroids and the use of a prolonged oral taper (six weeks) were instrumental in achieving full recovery and preventing the relapses often associated with abrupt steroid cessation [13].

The negative viral studies for HHV-6, EBV, and CMV do not exclude DRESS, as viral reactivation is not universally present [14,15] and may be detected later in the disease course, coinciding with clinical flares [16].

The neurological symptoms observed in our patient, including paresthesia (peripheral neuropathy) and dysgeusia (cranial nerve involvement), merit specific discussion. While less common than cutaneous or hepatic manifestations, neurological involvement in DRESS is increasingly recognized in the literature. A comprehensive review by Calle et al. notes that neurological symptoms in DRESS may include headaches, seizures, cranial nerve palsies, and muscle weakness [4]. Peripheral neuropathy with paresthesia has been documented in multiple DRESS cases. Valente et al. described a patient with carbamazepine-induced DRESS who presented with peripheral neuropathy manifesting as numbness and paresthesia [17]. Similarly, Vasanthan et al. reported a child with DRESS who developed peripheral neuropathy during the illness course, which improved with symptomatic treatment [18]. More severe neurological involvement, including central nervous system vasculitis, has also been described. Mesec et al. reported a case of carbamazepine hypersensitivity syndrome presenting as vasculitis of the CNS, demonstrating that carbamazepine-induced DRESS can affect both peripheral and central nervous systems [19]. Importantly, as noted by Gaha et al., cerebral vasculitis-like lesions in DRESS can be reversed completely by withdrawing the causal medication and instigating timely corticosteroid treatment [20]. In our patient, the prompt resolution of both paresthesia and dysgeusia with corticosteroid therapy and carbamazepine discontinuation supports their attribution to DRESS rather than a primary autoimmune process and aligns with the literature demonstrating reversibility of neurological manifestations with appropriate treatment [4,18,19,20].

For rheumatologists, this case is highly relevant. DRESS represents a severe model of immune dysregulation triggered by an exogenous agent. Its management - involving immunosuppression, slow tapering of steroids, and monitoring for sequelae - parallels the treatment of many autoimmune conditions. Furthermore, physicians across specialties, including rheumatology, dermatology, and internal medicine, frequently prescribe medications with known DRESS risk (e.g., sulfasalazine, allopurinol, antibiotics) and must be adept at recognizing this life-threatening complication [2,4,6,7].

Differential diagnoses considered

Given the multisystem presentation, alternative diagnoses were carefully considered. Drug-induced lupus erythematosus (DILE) was a consideration, particularly given the patient's long-term hydralazine use. However, DILE typically presents with arthralgias, myalgias, serositis, and positive antinuclear antibodies, which were absent in this case. Eosinophilia is not a hallmark of DILE. Drug-Induced vasculitis was another consideration, particularly given the neurological symptoms (paresthesia). However, the absence of palpable purpura, mononeuritis multiplex, or other vasculitis stigmata, along with the rapid resolution of symptoms with corticosteroid therapy and drug withdrawal, made primary vasculitis less likely. Furthermore, the temporal relationship with carbamazepine initiation, the presence of eosinophilia, and the systemic organ involvement (hepatic, pulmonary) align most closely with DRESS syndrome. We acknowledge that autoimmune serologies and complement levels were not obtained during admission, which represents a limitation of this retrospective case report.

## Conclusions

This case of carbamazepine-induced DRESS syndrome, initially misdiagnosed as ACEi angioedema, serves as a critical reminder of the complexities in diagnosing severe drug hypersensitivity reactions. It emphasizes that a detailed chronological medication history is essential, particularly focusing on agents started within the critical two-to-eight-week window prior to symptom onset. Clinicians must maintain a high index of suspicion for DRESS when a patient presents with a rash accompanied by systemic symptoms or hematologic abnormalities, especially if the clinical picture worsens or evolves after the withdrawal of an initially suspected drug. Prompt diagnosis, immediate cessation of the offending agent, and timely initiation of immunomodulatory therapy are vital to reducing morbidity and mortality associated with this life-threatening condition. 
